# Inhibition of TRPM7 suppresses cell proliferation of colon adenocarcinoma in vitro and induces hypomagnesemia in vivo without affecting azoxymethane-induced early colon cancer in mice

**DOI:** 10.1186/s12964-017-0187-9

**Published:** 2017-08-15

**Authors:** Junhao Huang, Hideki Furuya, Malika Faouzi, Zheng Zhang, Mahealani Monteilh-Zoller, F. Kelly Galbraith Kawabata, David Horgen, Toshihiko Kawamori, Reinhold Penner, Andrea Fleig

**Affiliations:** 1 Center for Biomedical Research, The Queen’s Medical Center, John A. Burns School of Medicine, University of Hawaii, 1301 Punchbowl St., Honolulu, HI 96813 USA; 2 Cancer Biology Program, University of Hawaii Cancer Center, 701 Ilalo St, Honolulu, HI -96813 USA; 3 Laboratory of Marine Biological Chemistry, Department of Natural Sciences, Hawaii Pacific University, Kaneohe, HI 96744 USA; 4 Present Address: Guangdong Provincial Key Laboratory of Sports and Health Promotion, Scientific Research Center, Guangzhou Sport University, Guangzhou, China; 5 Present Address: Department of Pharmacology, School of Pharmaceutical Sciences, Central South University, Changsha, Hunan China; 6 Present Address: Chikusa Central Clinic, Imaike, Chikusa-ku, Nagoya, Aichi Pref Japan

**Keywords:** Dysplastic aberrant crypt foci, Colon cancer, TRPM7, Mg^2+^ (magnesium) channel, Waixenicin A

## Abstract

**Background:**

Magnesium (Mg^2+^) is an essential cation implicated in carcinogenesis, solid tumor progression and metastatic potential. The Transient Receptor Potential Melastatin Member 7 (TRPM7) is a divalent ion channel involved in cellular and systemic Mg^2+^ homeostasis. Abnormal expression of TRPM7 is found in numerous cancers, including colon, implicating TRPM7 in this process.

**Methods:**

To establish a possible link between systemic magnesium (Mg^2+^) status, the Mg^2+^ conducting channel TRPM7 in colon epithelial cells, and colon carcinogenesis, in vitro whole-cell patch clamp electrophysiology, qPCR, and pharmacological tools were used probing human colorectal adenocarcinoma HT-29 as well as normal primary mouse colon epithelial cells. This was extended to and combined with aberrant crypt foci development in an azoxymethane-induced colorectal cancer mouse model under hypomagnesemia induced by diet or pharmacologic intervention.

**Results:**

We find that TRPM7 drives colon cancer cell proliferation in human HT-29 and expresses in normal primary mouse colon epithelia. This is linked to TRPM7’s dominant role as Mg^2+^ transporter, since high extracellular Mg^2+^ supplementation cannot rescue inhibition of cell proliferation caused by suppressing TRPM7 either genetically or pharmacologically. In vivo experiments in mice provide evidence that the specific TRPM7 inhibitor waixenicin A, given as a single bolus injection, induces transient hypomagnesemia and increases intestinal absorption of calcium. Repeated injections of waixenicin A over 3 weeks cause hypomagnesemia via insufficient Mg^2+^ absorption by the colon. However, neither waixenicin A, nor a diet low in Mg^2+^, affect aberrant crypt foci development in an azoxymethane-induced colorectal cancer mouse model.

**Conclusion:**

Early stage colon cancer proceeds independent of systemic Mg^2+^ status and TRPM7, and waixenicin A is a useful pharmacological tool to study of TRPM7 in vitro and in vivo.

**Electronic supplementary material:**

The online version of this article (doi:10.1186/s12964-017-0187-9) contains supplementary material, which is available to authorized users.

## Background

Magnesium (Mg^2+^) is the second-most abundant divalent ion present in mammalians [1]. Cell proliferation needs the presence of Mg^2+^ [2]. Malignant tissues require elevated levels of energy and Mg^2+^ [3], accumulating Mg^2+^ at the expense of surrounding tissues and acting as Mg^2+^ traps [1]. In vivo experiments have revealed a reduction of primary tumor growth and neoangiogenesis in Mg^2+^-deficient mice [4], which questions the current standard care in chemotherapy of Mg^2+^ replacement during cisplatin-induced hypomagnesemia [5]. Oncologists have noted the connection between hypomagnesemia and cancer [6, 7], although the role of Mg^2+^ in carcinogenesis remains controversial despite substantial clinical and epidemiological research [8]. Recent evidence indicates that Mg^2+^-transporting ion channels, specifically the ubiquitously expressed TRPM7 channel, could be critically involved in cancer biology [3].

TRPM7 is a member of the melastatin-like transient receptor potential (TRPM) subfamily, and a unique protein combining an ion channel with a functional α-kinase domain [9]. TRPM7, and its sister protein TRPM6, are the only two known channels conducting Ca^2+^, Mg^2+^, and trace metals into cells [10]. While the channel domain regulates systemic Mg^2+^ in mammalian organisms and maintains cellular Mg^2+^ concentrations [11], the kinase domain is involved in sensing Mg^2+^ status [12]. Knock-down of TRPM7 results in cellular growth arrest, which is reversible by high extracellular Mg^2+^ supplementation [13]. Additional studies implicate TRPM7 in tumor growth: the channel is abundantly expressed in a variety of human carcinoma cells and TRPM7 deficiency suppresses their growth [14]. In human nasopharyngeal carcinoma, the channel has been implicated in carcinogenesis [15]. Overexpression of TRPM7 is detected in breast cancer tissues correlating with their proliferative potential [16]. TRPM7 is also implicated as a player in colon-related chemoresistance [17], as colon carcinoma LoVo cells that remain sensitive to doxorubicin treatment exhibit higher TRPM7 expression compared to their drug-resistant counterparts. TRPM7’s role in differentiated, non-proliferating tissue seems to be at variance from that seen in proliferative cell types; For example, suppression of TRPM7 protects hippocampal neurons from death in ischemia [18]. Thus, interference with TRPM7’s physiological function may be more detrimental to proliferating cell types. Since dedifferentiation status of cancerous tissue is linked to the aggressiveness of tumor growth, therapeutic intervention targeting TRPM7 may have significant impact on highly malignant tumors. Finally, epidemiologic studies link a genetic polymorphism in TRPM7 to risk of developing colorectal neoplasia, particularly in subjects with a high calcium:magnesium dietary ratio [19].

## Methods

### Cell culture

The human colon epithelial cell-line HT-29 from colon adenocarcinoma patient was acquired from ATCC®, USA (ATCC® HTB38™). ATCC® utilizes Short Tandem Repeat (STR) PCR assay profiling for cell line authentication. HT-29 were grown in McCoy’s 5A Medium (Cellgro, USA) containing 10% FBS (Gibco, USA) at 37 °C in a humidified atmosphere of 5% CO_2_ in air. Tetracycline-inducible HEK293-TREx cells stably expressing human TRPM7 (hTRPM7) as confirmed by whole-cell electrophysiology were cultured and treated as previously described [13].

### Primary culture of mouse colon epithelial cells (MCECs)

Details of primary culture of MCECs were previously described [20]. Briefly, mouse colon epithelial tissue was isolated from 8-week old C57BL/6 J mouse. Post extraction, tissue was mechanically minced, then enzymatically digested in DMEM containing 75 U/ml collagenase, 20 μg/ml dispase neutral protease and 1% FBS for 2 h. Colon crypts selected from digested tissue via sedimentation with 0.11 M of D-sorbitol were grown in collagen-coated 12-well plates under a humidified atmosphere of 5% CO_2_ at 37 °C with DMEM which contained 4.5 g/L glucose, 0.68 M sodium pyruvate, 2.5% FBS, 0.25 U/ml insulin, 100 U/ml benzyl penicillin, 30 μg/ml streptomycin sulfate and 25 μg/ml gentamicin. 50% of the culture media were replaced every 2–3 days.

### Proliferation assay

Cells were seeded at a density of 10^5^ cells/ml in medium and allowed to attach before chemicals were added. Cells were incubated at 37 °C in a humidified incubator for experiments. To prepare medium containing 50 μM Mg^2+^, DMEM without Mg^2+^ and Ca^2+^ was used. MgCl_2_ was added to make it to the final concentration of 50 μM. CaCl_2_ was added to adjust to the regular medium concentration of 900 μM. No EDTA was added. A Beckman Coulter ViCell Automated Cell Viability Analyzer (Beckman Coulter, USA) was used for cell counting as previously described [21].

### Electrophysiology

Patch-clamp experiments were performed at room temperature (20 ± 2 °C) using the whole-cell configuration as previously described [22]. High-resolution currents were acquired and recorded by EPC-10 (HEKA, Germany) and PatchMaster software (HEKA, Germany). All voltages were corrected for a liquid junction potential of 10 mV between external and internal solutions. Currents were elicited by ramps of 50 ms from −100 mV to +100 mV acquired at 0.5 Hz and a holding potential of 0 mV. Currents at +80 mV were extracted, normalized to cell size, and plotted versus time of the experiment. Standard extracellular solution contained (in mM): 140 NaCl, 1 CaCl_2_, 2.8 KCl, 2 MgCl_2_, 10 HEPES-NaOH and 11 Glucose (pH 7.2; 300 mOsm). Mg^2+^-free intracellular solution contained (in mM): 120 Cs-glutamate, 8 NaCl, 10 HEPES and 10 Cs-BAPTA (pH 7.2; 300 mOsm). For Mg^2+^ dose-response assessment the intracellular solution contained (in mM): 120 Cs-glutamate, 8 NaCl, 10 HEPES and 10 Cs-BAPTA plus appropriate amounts of MgCl_2_, as calculated with WebMaxC Standard (Chris Patton, Stanford, USA). Overexpressed hTRPM7 currents in HEK293-TREx cells were assessed after 18 h to 24 h of tetracycline exposure.

### Small interfering RNA silencing

Cells were transfected with 20 nM siRNA designed by Life Technologies and Lipofectamine RNAiMAX Reagent (Life Technologies, USA) according to the manufacturer’s instructions. siRNAs for silencing of the target genes were as follows: TRPM6siRNA-5′-CGCUAUCGCUACAUCAUGATT-3′; and TRPM7siRNA-5′-GAUUUGCACUAUCGGAAUATT-3′. A negative scrambled siRNA (Life Technologies, USA) was used. Cells were used 72 h after siRNA transfection.

### RT-PCR analysis

Total RNA was isolated from HT-29 cells using RNeasy Mini Kit (Qiagen, USA). SuperScript III First-Strand Synthesis System for RT-PCR (Life Technologies, USA) was used following the manufacturer’s procedure to synthesize cDNA from 1 μg total RNA primed with oligo (dT)primers. Gene-specific primer pairs for TRPM6 (forward primer 5′- TGCCCTGGAACAAGCAATGTCAG -3′; reverse primer 5′- CTTTTCATCAGCACAGCCCAAACC -3′), TRPM7 (forward primer 5′- AGCATACAGAACAGAGCCCAACGG -3′; reverse primer 5′- TTCCAACAGTGCCATCATCCACC -3′), and GAPDH (forward primer 5′- GGAGCCAAAAGGGTCATCATCTC -3′, reverse primer 5′- AGTGGGTGTCGCTGTTGAAGTC -3′) were designed using MacVector and synthesized by Life Technologies. PCR was performed in reaction volumes of 50 μl containing 1 μl dNTPs (10 mM), 2 μl each primer (10 pmol/μl), 2 μl cDNA solution, 5 μl 10× reaction buffer, 37 μl water, and 1 μl Pfu Ultra II fusion HS DNA polymerase (Stratagene, USA) on a Thermal Cycler (BioRad, USA). Denaturation was carried out at 94 °C for 20 s, annealing at 55 °C for 30 s and elongation at 72 °C for 30 s for 35 cycles, followed by extension at 72 °C for 3 min. PCR products were detected in 0.8% agarose gel containing 1× SYBR Safe DNA Gel Stain (Life Technologies, USA).

### Q-PCR

q-PCR was performed to examine the mRNA expression levels of TRPM6, TRPM7, SLC41A1, SLC41A2, SLC41A3, MagT1, NIPA1, N33, CNNM1 and CNNM2 by using β-actin as a reference for normalization. Total RNA (1 μg) was extracted from HT-29 cells using the RNeasy Mini Kit (Qiagen, USA). The random priming was utilized to convert mRNA to cDNA by ABI’s High Capacity cDNA RT Kit with RNase Inhibitor (Life Technologies, USA). The q-PCR was performed using the ABIs’ HT7900 FAST Real-Time PCR System (Life Technologies) and the ABI’s POWER SYBRGreen (Life Technologies, USA) according to the manufacturers’ instructions. Gene-specific primer pairs of human TRPM6, TRPM7, SLC41A1, SLC41A2, SLC41A3, MagT1, NIPA1, N33, CNNM1, CNNM2, GAPDH and β-actin were purchased from Qiagen.

### Preparation of waixenicin A

For in vitro assays, 25 μg of purified and lyophilized waixenicin A (waixA) was dissolved in 25 μl of methanol and diluted in the adequate buffer solution to give 100 μM stock solution. The stock solutions were made fresh just prior to each assay. For in vivo assays, immediately prior to injection, purified and lyophilized waixA was dissolved in Cremophor EL® /ethanol (1:1) at 8 or 50 mg/ml. The solution was diluted 1:9 in saline and sterile filtered giving final concentrations of 0.80 or 5.0 mg/ml of waixenicin A in 5% Cremophor EL, 5% ethanol that were injected at appropriate volumes to achieve 8 or 25 mg/kg, respectively. Vehicle was prepared by diluting Cremophor EL® /ethanol (1:1) 1:9 in saline and sterile filtering.

### Animals

All animal experiments were performed in compliance with the animal ethics board of the University of Hawaii. 8-week-old male C57BL/6 mice were purchased from Charles River Laboratories. In low Mg^2+^ diet experiments, mice were fed the control (AIN-93 M, 0.05% Mg^2+^) or Mg^2+^-oxide deficient (AIN-93 M, 0.003% Mg^2+^) diet and sacrificed by CO_2_ exposure followed by cervical dislocation at days 7, 14, 21 and 28. Mice were transferred to metabolic cages and 24-h urine and feces were collected on the last day before sacrifice. For serum collection, blood was obtained by puncturing the heart with a syringe without clotting factors and processed the following way: at least 500 μl of blood were harvested and centrifuged for 15 min. at 1500×g. The supernatant (serum) was stored at −20 °C until use. Bone (femur) samples were collected. In waixA treatment, mice were intraperitoneally (i.p.) injected once with vehicle (5% Cremophor EL in phosphate buffered saline (PBS)) or waixA (25 mg/kg) in vehicle, and sacrificed on days 1, 2, 3, 5, 7 and 14 after injection. Injection volume was 10 μl per g of body weight.

In azoxymethane (AOM)-induced aberrant crypt foci (ACF) formation experiments, 8-week-old male C57BL/6 mice were fed the control or Mg^2+^-deficient diet for 2 weeks. Mice were then i.p. injected with PBS or AOM (10 mg/kg) in PBS once a week for 3 weeks and sacrificed 2 weeks after the last AOM injection. Mice were transferred to metabolic cages and 24-h urine and feces were collected on the last day before sacrifice. Blood and bones were collected. The method for the Aberrant Crypt Foci (ACF) formation assay was described previously [23]. Briefly, after laparotomy, the entire colon was resected and filled with 10% of neutral buffered formalin, opened longitudinally from the anus to the cecum and fixed with 10% of neutral buffered formalin for 24 h. All colons were then stained with 0.1% methylene blue in saline and counted for the number of ACF under a light microscope following the procedure of Bird [24].

In the combined waixA and AOM treatment experiments, mice fed the control diet were i.p. injected with PBS or AOM (10 mg/kg) in PBS once a week for 3 weeks and sacrificed 2 weeks after the last AOM injection. Mice received i.p. injection of vehicle (5% Cremophor EL in PBS) or waixA (8 mg/kg) in vehicle 1 day before AOM injection once a week for 3 weeks. Mice samples (serum, bone, feces, and urine) were collected for Mg^2+^ analysis. In the combined low Mg^2+^ diet and AOM treatment experiment, mice were fed the control (AIN-93 M, 0.05% Mg^2+^) or Mg^2+^-oxide deficient (AIN-93 M, 0.003% Mg^2+^) diet. Control or low Mg^2+^ diet mice were injected i.p. with PBS or AOM (10 mg/kg) once a week for 3 weeks and sacrificed 2 weeks after the last AOM injection.

### Mg^2+^ and Ca^2+^ measurements in mice samples

Blood samples were spun down for 15 min at 1500×g and supernatant (serum) was collected for Mg^2+^ and Ca^2+^ analysis. Feces were dried for 8 h at 110 °C, incinerated for 5 h at 625 °C and resuspended in 12% HCl overnight. The next day, the fecal solution was spun down for 15 min at 1500 rpm, and the supernatant was used for the analytical procedures. Bones were incinerated for 8 h at 500 °C and resuspended with 1:10 *w*/*v* of concentrated HNO_3_-H_2_O_2_ (2:1), on a heating plate until complete discoloration was achieved. The mineral solution was dissolved with 1:10 *w*/*v* of 2% aqueous HNO_3_. The Mg^2+^ and Ca^2+^ content of serum, urine, feces and bones was measured by Varian Vista MPX inductively coupled plasma optical emission spectrophotometer (ICPOES).

### Statistical analysis

Patch-clamp data were acquired with PatchMaster software and exported to IGOR Pro (Wavemetrics). Current amplitudes were extracted from IGOR Pro and transferred to Excel (MS Office 2007) where all values for mean and standard error of the mean (SEM) were calculated. In some cases *p*-values based on 2-tail, unpaired Student’s T-tests, assuming unequal variance between populations, were calculated (Excel, TTEST function). For cell proliferation experiments, 2-tail paired Student’s T-tests were used for significance analysis and *p* < 0.05 was considered statistically significant.

## Results

### Channel kinase expression and endogenous TRPM7-like currents in HT-29

**Fig. 1:**
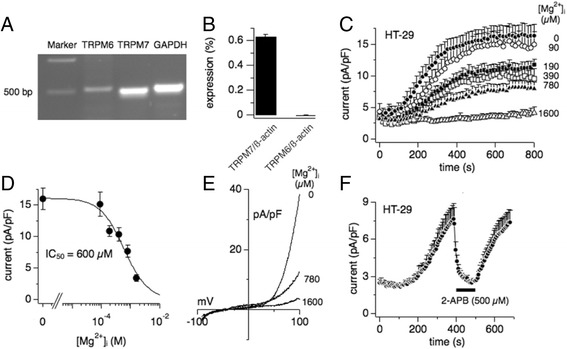
Endogenous TRPM7-like currents in HT-29. **a** RT-PCR analysis of TRPM6 and TRPM7 mRNA expression. GAPDH was used as the housekeeping gene. **b** Percentage expression of TRPM6 or TRPM7 mRNA to the housekeeping gene β-actin was represented by q-PCR analysis (*n* = 3). **c** Average normalized time course of TRPM7-like current development in HT-29 at various [Mg^2+^]_i_ (*n* = 6–12). Decrease of [Mg^2+^]_i_ from 1600 to 0 μM allowed TRPM7-like current development in a dose-dependent manner. **d** Dose-response curve for [Mg^2+^]_i_ from data in C. Currents were extracted at +80 mV at 800 s, normalized to cell size in pA/pF, averaged and plotted against [Mg^2+^]_i_, and approximated by dose-response curve fit (IC_50_ = 600 μM; Hill coefficient = 1). **e** Current-voltage (I/V) relationships f TRPM7-like currents from representative HT-29 cells for [Mg^2+^]_i_ at 0, 780 and 1600 μM and extracted at 800 s. **f** Average normalized time course of TRPM7-like current development and inhibition by 500 μM 2-APB as indicated by the *black bar* ([Mg^2+^]_*i*_ = 0 μM; *n* = 7)

To establish a possible link between TRPM6, TRPM7, magnesium (Mg^2+^) and colon carcinogenesis, we first set out to characterize channel kinases in vitro using the human colorectal adenocarcinoma cell line HT-29. Both TRPM6 and TRPM7 are expressed in colon tissue [9, 25] and RT-PCR confirmed the expression of both TRPM6 and TRPM7 in HT-29 cells (Fig. 1a). However, quantitative PCR analysis demonstrated a 35-fold higher expression level of TRPM7 mRNA compared to TRPM6 mRNA (Fig. 1b). 2-Aminoethyl diphenylborinate (2-APB) is known to inhibit TRPM7, to facilitate TRPM6 and to have reduced efficacy on heteromeric TRPM6/TRPM7 currents as assessed by whole-cell patch-clamp recordings [26, 27]. Figure 1f shows that extracellular application of 500 μM 2-APB completely blocked the fully developed TRPM7-like whole-cell currents in HT-29 cells. It is well established that channel kinase activity is negatively regulated by intracellular Mg^2+^ [9]. A dose-response curve of increasing intracellular Mg^2+^ supplementation using the whole-cell patch clamp technique revealed an IC_50_ of 600 μM for TRPM7-like currents in HT-29 cells, in line with TRPM7-like currents assessed in other native cell line systems (Fig. 1c, d; [28, 29]). Figure 1e shows representative current-voltage (I/V) relationships of native TRPM7-like currents in HT-29 cells in the absence or with Mg^2+^ supplementation of the internal solution (see Methods). Together, these data suggest that TRPM7 carries the majority of TRPM7-like whole-cell currents in HT-29.

### Waixenicin A Mg^2+^-dependently inhibits native TRPM7-like currents in HT-29

**Fig. 2:**
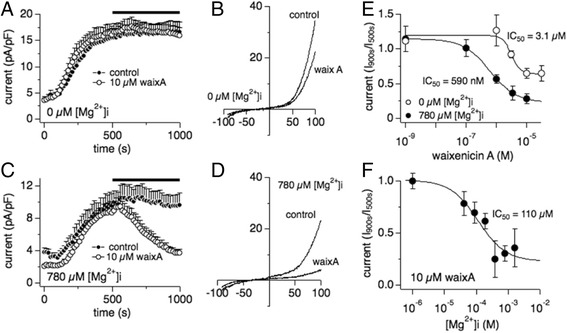
Waixenicin A Mg^2+^-dependently blocks endogenous TRPM7-like currents in HT-29. **a** Averaged normalized time course of TRPM7-like current development in the presence of 0 μM [Mg^2+^]_i_ without (*n* = 11) and with 10 μM waixenicin A (waixA) application (*n* = 10) as indicated by the *black bar*. **b** I/V relationships from a representative cell obtained at 1000 s and taken from data in (**a**). **c** Averaged normalized time course of TRPM7-like current development in the presence of 780 μM [Mg^2+^]_i_ without (*n* = 10) and with 10 μM waixA application as indicated by the *black bar* (*n* = 10). **d** I/V relationships from representative cells obtained at 1000 s and taken from data in (**c**). **e** Dose-response curve for inhibition of TRPM7-like currents by increasing concentrations of externally applied waixA in 0 μM or 780 μM [Mg^2+^]_i_. Currents were extracted at +80 mV at 900 s, normalized to current at 500 s, plotted against waixA concentrations, and approximated by a dose-response curve fit. **f** Dose-response curve for [Mg^2+^]_i_ with externally applied 10 μM waixA at 500 s and for 500 s. Currents were extracted at +80 mV at 900 s, normalized to current at 500 s, plotted against [Mg^2+^]_i_, and approximated by a dose-response curve fit (*n* = 4–10)

Waixenicin A (waixA) is a Mg^2+^-dependent specific inhibitor of TRPM7, but not TRPM6 [21, 27]. In the absence of intracellular Mg^2+^ in the internal solution ([Mg^2+^]_i_), extracellular application of 10 μM waixA exerted only a weak effect on TRPM7 whole-cell currents (Fig. 2a & b). A dose-response analysis of waixA-mediated inhibition of TRPM7 revealed an IC_50_ of 3.1 μM without reaching full inhibition of the current (Fig. 2e, open circles). On the other hand, in the presence of physiological 780 μM [Mg^2+^]_i_, 10 μM of waixA strongly inhibited TRPM7 currents (Fig. 2c, d & e). The IC_50_ was shifted from 3.1 μM in zero [Mg^2+^]_i_ to 590 nM in 780 μM [Mg^2+^]_i_ (Fig. 2e). To test the Mg^2+^-dependence of waixA in HT-29 cells, we next applied 10 μM waixA at various [Mg^2+^]_i_. A concentration of 10 μM waixA Mg^2+^-dependently blocked TRPM7 current with an IC_50_ of 110 μM of [Mg^2+^]_i_ (Fig. 2f). These data confirm waixA as a potent TRPM7 inhibitor in HT-29 cells in the presence of intracellular Mg^2+^.

### TRPM7 siRNA and waixenicin a suppresses cell proliferation of HT-29

**Fig. 3:**
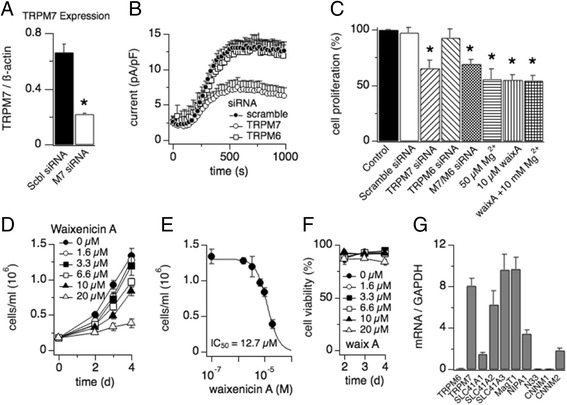
Waixenicin A inhibits cell proliferation of HT-29. **a** q-PCR analysis represented the ratio of TRPM7 mRNA to the housekeeping gene β-actin 72 h after HT-29 cell transfection with 20 nM of TRPM7 siRNA or scramble siRNA. *p < 0.01 vs. scramble siRNA. *n* = 3 experiments. **b** Endogenous TRPM7-like currents at +80 mV 72 h after HT-29 cell transfection with 20 nM of TRPM7 (open circles, *n* = 8), TRPM6 (open squares, *n* = 6) or scramble siRNA (filled circles, *n* = 8). **c** Mg^2+^ involvement in proliferation of HT-29. Cell proliferation was examined at 72 h after incubation of scramble siRNA, TRPM7 siRNA, TRPM6 siRNA, TRPM7 siRNA combined with TRPM6 siRNA, 50 μM Mg^2+^, or 10 μM waixA with or without the presence of 10 mM Mg^2+^ (*p < 0.05 vs. Control, *n* = 3). **d** Normalized total cell count of HT-29 treated with different concentrations of waixA for various periods of time and analyzed via ViCell assay (*n* = 3). **e** Dose-response curve for total cell count at day 4 with different concentrations of waixA (IC_50_ = 12.7 μM; Hill coefficient = 1.67). Total cell count of untreated control represents 100% of proliferation. **f** Cell viability in dependence of increasing waixA concentrations as assessed using the MTT assay (*n* = 3). **g** Expression profile of putative Mg^2+^ transporters in HT-29 cells established by q-PCR (*n* = 3)

To determine the involvement of TRPM7 in colon cancer cell proliferation, we next performed RNA interference (RNAi). A 21-nucleotide siRNA duplex specific to human TRPM7 was used. Our q-PCR analysis showed a 67% decrease in TRPM7 mRNA in cells transfected with TRPM7 siRNA compared to scrambled siRNA control (Fig. 3a; *p* < 0.01). To examine the effect of TRPM7 siRNA on TRPM7 currents, we performed patch-clamp recordings in HT-29 cells 72 h after TRPM7 siRNA transfection. As seen in Fig. 3b, TRPM7-like currents were decreased by 60% in cells transfected with TRPM7 siRNA compared to scramble siRNA control (*p* < 0.001). However, transfection with TRPM6 siRNA had no effect on TRPM7-like current (Fig. 3b).

We next tested whether the activity of TRPM7 influenced the cell growth of HT-29. As seen in Fig. 3c, cell proliferation was decreased by 35% in cells transfected with TRPM7 siRNA compared to scrambled siRNA control (*p* < 0.05). However, transfection with TRPM6 siRNA had no effect on cell proliferation (Fig. 3c). Co-transfection of cells with TRPM7 and TRPM6 siRNA significantly inhibited cell proliferation (70 ± 3.8%; *p* < 0.05). This effect showed no significant difference between transfection of TRPM7 siRNA alone (65 ± 7.4%). Consistent with our mRNA analysis and electrophysiological recording, this result indicates that TRPM7 but not TRPM6 plays an important role in HT-29 cell proliferation.

The data in Fig. 2 show that waixA blocks TRPM7 currents. We therefore tested the effect of waixA on cell proliferation in HT-29 cells. Incubation of cells with different concentrations of waixA for various periods of time reduced the total number of cells in a dose-dependent manner (Fig. 3d). The dose-response analysis of waixA-mediated inhibition of cell proliferation revealed an IC_50_ of 12.7 μM (Fig. 3e). WaixA did not affect cell viability, even at 20 μM concentrations (Fig. 3f). Consistent with our previous study [21], cell cycle analysis using flow cytometry showed that waixA suppressed HT-29 cell proliferation by preventing cells from entering the synthesis (S) phase (see Additional file 1). As the FACS analysis in Additional file 1: Figure S1 shows, waixA dose-dependently decreased cell numbers in the S-phase by about 25%, whereas it increased the number of cells in the G0/G1-phase by about 20%, and G2/M-phase by about 10%.

TRPM7 is a Mg^2+^-transporting channel and plays an important role in cellular Mg^2+^ regulation [9, 12]. Inhibition of TRPM7 reduces cellular Mg^2+^ contents and suppresses cell proliferation [13]. As shown in Fig. 3c, reduction of extracellular Mg^2+^ concentration ([Mg^2+^]_o_) from 2 mM to 0.05 mM significantly inhibited the cell proliferation of HT-29 (*p* < 0.05), which was similar to the effect of 10 μM waixA (55.7 ± 9.01% vs. 55.0 ± 4.76%, respectively; *p* > 0.05). It has been shown that the growth defect observed in TRPM7-deficient cells can be rescued by high extracellular Mg^2+^ supplementation via compensatory Mg^2+^ uptake pathways [30]. We therefore asked the question whether the inhibitory effect of waixA on HT-29 cell proliferation could be reversed by supplementation of 10 mM extracellular Mg^2+^. As seen in Fig. 3c, 10 mM Mg^2+^ with 10 μM waixA was unable to rescue cell proliferation compared to incubation with 10 μM waixA alone (55.0 ± 4.76% vs. 54.2 ± 4.77%, respectively; *p* > 0.05). This might be explained by the low abundance or lack of alternative Mg^2+^ uptake mechanisms in HT-29. We therefore performed q-PCR analysis of Mg^2+^ transporters in HT-29 (Fig. 3g). Although SLC41A2, SLC41A3 and MagT1 showed comparable expression levels, they were not able to compensate siRNA-induced knockdown of TRPM7. SLC41A1, NIPA1, N33 and both CNNM1 and 2 were either not detected or expressed at very low levels. Taken together, our results indicate that TRPM7 appears to represent the major Mg^2+^-transporting mechanism in colon cancer HT-29 cells.

### Waixenicin A blocks TRPM7-like current in primary mouse colon epithelial cells

**Fig. 4:**
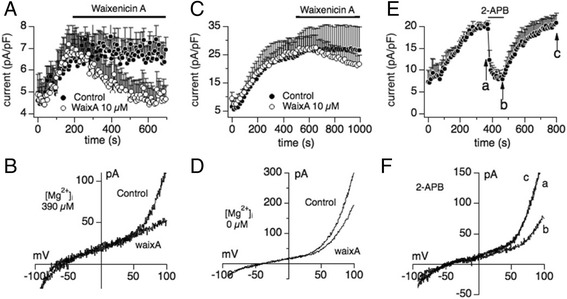
Waixenicin A blocks endogenous TRPM7-like currents in primary mouse colon epithelial cells. Primary mouse colon epithelial cells (MCECs) were established as described in the method section and investigated by whole-cell patch clamp technique for inhibition by waixA and 2-APB. **a** Average normalized time course of TRPM7 current development in MCECs in the presence of 390 μM [Mg^2+^]_i_ without (*n* = 5) and with 10 μM waixA external application as indicated by the *black bar* (*n* = 6). **b** I/V relationships from a representative cell obtained at 1000 s from data in (**a**). **c** Average normalized time course of TRPM7-like current development in MCECs in the presence of 0 μM [Mg^2+^]_i_ without (*n* = 6) and with 10 μM waixA application (*black bar*; *n* = 5). **d** I/V relationships from a representative cell obtained at 500 s from data in (**c**). **e** Average normalized time course of TRPM7-like currents. At the time indicated by the *black bar*, cells were superfused with external solution supplemented with 100 μM 2-APB (*n* = 4; [Mg^2+^]_*i*_ = 0 μM). **f** I/V relationships from a representative cell displayed before (**a**) or after (**b**) 2-APB application and after 2-APB washout (**c**)

To confirm the relevance for our in vivo carcinogenesis studies, we determined the pharmacological profile of native TRPM7-like currents in primary mouse colon epithelial cells (MCECs; see Methods; (20)). In the presence of physiological 390 μM [Mg^2+^]_i_, 10 μM waixA strongly inhibited TRPM7 currents in MCECs (Fig. 4a & b). As expected, 10 μM of waixA had only a weak inhibitory effect on TRPM7-like currents in the absence of [Mg^2+^]_I_ (Fig. 4c & d). We next tested 2-APB to determine whether TRPM7 would constitute the major protein underlying TRPM7-like whole-cell currents in MCECs. As seen in Fig. 4e and f, extracellular application of 100 μM 2-APB completely blocked TRPM7-like currents. This pharmacological profile suggests that TRPM7, rather than TRPM6, carries this current.

### Mg^2+^-deficient diet induces hypomagnesemia in mice

**Fig. 5:**
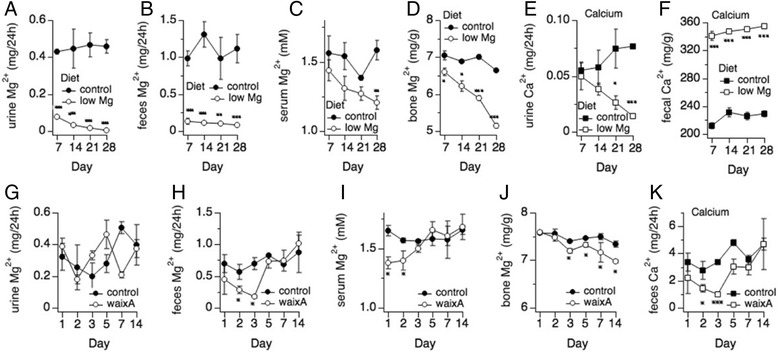
Waixenicin A transiently mimics the impact of a Mg^2+^-deficient diet on systemic Mg^2+^ and Ca^2+^ homeostasis in vivo. **a**-**d**: Mg^2+^ concentrations in urine, feces, serum and bones from mice fed control (0.05% Mg^2+^) or Mg^2+^-deficient (0.003% Mg^2+^) diet. Mice were sacrificed on day 7, 14, 21, and 28 after feeding. Serum, urine, feces and bone were collected and assessed for Mg^2+^ (*p < 0.05, **p < 0.01, ***p < 0.001 vs. 0.05% Mg^2+^ diet; *n* = 3–6). **e**-**f** Ca^2+^ concentrations assessed in urine and feces from mice in (**a**-**d**). No changes were found in Ca^2+^ levels for serum and bone (data not shown). **g**-**j** Mg^2+^ concentrations in urine, feces, serum and bone from mice intraperitoneally injected once with vehicle (PBS) or waixA (25 mg/kg). Mice were sacrificed on day 1, 2, 3, 5, 7 and 14 after injection. Samples were collected for Mg^2+^ and Ca^2+^ analysis (*p < 0.05 vs. vehicle. *n* = 3). **k** Ca^2+^ concentrations in feces from same mice as in (**g**-**j**) (*p < 0.05; ***p < 0.001 vs. vehicle; *n* = 3). No statistically significant difference was found for Ca^2+^ levels in the samples from urine, serum, or bone (data not shown)

TRPM7 is emerging as a key player in cancer growth, migration and invasion. Increased expression levels of TRPM7 have been associated with poor prognosis in several cancers [14]. Furthermore, data from a mouse xenograft tumor model show that magnesium deficiency inhibits primary tumor growth while favoring metastasis [4]. We wondered whether hypomagnesemia would prove protective in the development of colorectal cancer, and whether this could be linked to TRPM7 activity. To this end we first confirmed that 8-week-old C57BL mice would not only develop diet-induced hypomagnesemia but also survive when fed with control (0.05% Mg^2+^) or Mg^2+^-deficient (0.003% Mg^2+^) diet for up to 28 days. Mice were transferred into metabolic cages for 24 h before sacrifice, after which serum, urinary, fecal and bone samples were collected and assessed for Mg^2+^ and Ca^2+^ concentrations. The results revealed that mice fed the Mg^2+^-deficient diet excreted significant less urine and feces in 24 h than mice fed control diet while maintaining normal body weight (Table 1). Subjecting mice to the Mg^2+^-deficient diet for 14, 21 and 28 days, also led to a significant decrease of Mg^2+^ concentration in serum, urine, fecal matter and bone compared to mice fed with the control diet (Fig. 5a-d), indicating that they had altered systemic Mg^2+^ homeostasis. Mice in Mg^2+^-deficient diet groups also developed lower levels of Ca^2+^ in urine (65% reduction for 21 days, *p* < 0.05; 81% reduction for 28 days, *p* < 0.001; Fig. 5e) while excreting significantly higher levels of Ca^2+^ through fecal matter (~50% increment for 7, 14, 21 and 28 days, *p* < 0.001; Fig. 5f), indicating that hypomagnesemia also altered Ca^2+^ homeostasis. However, no changes were found for Ca^2+^ levels in serum and bones when comparing control and Mg^2+^-deficient mice (data not shown). We conclude that a diet low in Mg^2+^ induces significant hypomagnesemia within 7 days, and that mice tolerate four weeks of significantly reduced Mg^2+^ intake.

**Table 1:** Physiological parameters from mice fed control or Mg^2+^-deficient diet

	Day 7		Day 14		Day 21		Day 28	
	0.05% Mg^2+^	0.003% Mg^2+^	0.05% Mg^2+^	0.003% Mg^2+^	0.05% Mg^2+^	0.003% Mg^2+^	0.05% Mg^2+^	0.003% Mg^2+^
Bodyweight, g	20.5 ± 0.38	20.9 ± 0.60	20.3 ± 0.50	21.6 ± 0.38	21.6 ± 0.19	22.1 ± 0.19	23.2 ± 0.79	23.1 ± 0.94
Urine, ml/24 h	1.8 ± 0.18	1.1 ± 0.011*	1.7 ± 0.38	0.7 ± 0.05**	1.5 ± 0.25	1.1 ± 0.19	1.9 ± 0.16	0.9 ± 0.27*
Feces, g/24 h	0.17 ± 0.017	0.09 ± 0.013**	0.20 ± 0.015	0.11 ± 0.008***	0.22 ± 0.012	0.11 ± 0.017**	0.22 ± 0.013	0.12 ± 0.015**

Mice were fed the control (0.05% Mg^2+^) or Mg^2+^-deficient (0.003% Mg^2+^) diet and sacrificed on 7, 14, 21 and 28 days. Mice were transferred to metabolic cages for 24 h. Bodyweight, excreted feces and urine per 24 h were measured. Results are displayed as mean ± S.E.M

*p < 0.05

**p < 0.01

*** *p* < 0.001 vs. 0.05% Mg^2+^ diet. *n* = 3–6

### Waixenicin A transiently disrupts intestinal Mg^2+^ and Ca^2+^ absorption and reduces bone Mg^2+^ levels

Mice with suppressed TRPM7 channel activity are hypomagnesemic [11]. We therefore hypothesized that systemic suppression of TRPM7 activity by waixA in vivo would similarly lead to a hypomagnesemic phenotype over time. To this end, 8-week old C57BL mice were fed with the control diet receiving a single bolus i.p. injection with either vehicle or waixA (25 mg/kg) on day zero. Mice were sacrificed after day 1, 2, 3, 5, 7, and 14. Serum, urinary, fecal and bone samples were collected for Mg^2+^ and Ca^2+^ analysis. The results show that, in comparison with mice of the vehicle group, mice treated with waixA had a significant decrease of Mg^2+^ concentration in serum for the first two days after injection, after which Mg^2+^ increased back to control levels (12.2% reduction, *p* < 0.05; Fig. 5i). In addition, fecal Mg^2+^ excretion in the waixA group was significantly lower compared to control up to day 3 (Fig. 5h; *p* < 0.01), indicating that waixA increased intestinal Mg^2+^ absorption during that time. However, Mg^2+^ excretion through urine was not affected (Fig. 5g). Finally, Mg^2+^ content in bone steadily decreased during the 14 days of observation (Fig. 5j; *p* < 0.05). Mice treated with waixA also had significantly reduced Ca^2+^ levels in feces (*p* < 0.05; Fig. 5k), however, serum, urine, and bone levels were unaffected (data not shown). Taken together, our result indicate that bolus application of waixA partially mimics diet-induced hypomagnesemia in that it transiently reduces Mg^2+^ serum levels and Mg^2+^ wasting through fecal matter, and causes loss of Mg^2+^ in bone within the observed time frame.

### Intestinal Mg^2+^ absorption is decreased in the early stage of colon cancer development

The time-course experiment using a bolus injection of waixA (Fig. 5) allowed us to design experiments that could determine whether this TRPM7 inhibitor would prove protective in an early-stage colon cancer mouse model. To this end, we used the well-established, chemically-induced colorectal cancer model with azoxymethane (AOM) to rapidly recapitulate the formation of aberrant crypt foci (ACF), pre-neoplastic lesions of adenocarcinoma [31]. Here, mice were divided in four arms with different i.p. injections: PBS; 5% Cremophor EL in PBS with waixA (8 mg/kg); PBS with AOM (10 mg/kg); and AOM (10 mg/kg) with waixA (8 mg/kg). The mice were fed the standard control diet. The two control groups were injected with either vehicle or vehicle with AOM once a week for 3 weeks and sacrificed 2 weeks after the last AOM injection. The two experimental groups were additionally injected with waixA the day before AOM injection, once a week for three weeks to support availability of waixA in the system. Serum, urinary, fecal, and bone samples were collected for Mg^2+^ analysis.

**Fig. 6:**
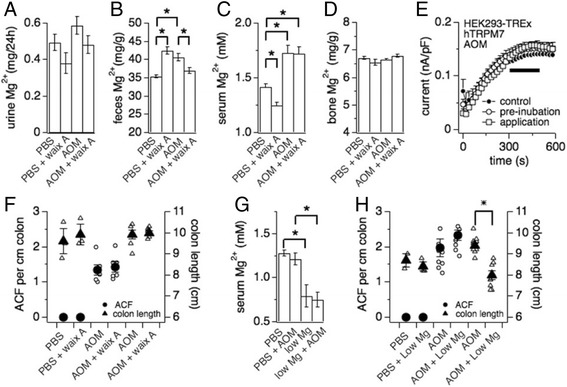
Waixenicin A inhibits intestinal Mg^2+^ absorption and causes hypomagnesemia without affecting aberrant cyst formation in AOM-treated mice. **a**-**d** Mice fed with the control diet were intraperitoneally (i.p.) injected with vehicle (*n* = 4), vehicle and waixenicin A (waixA; 8 mg/kg; *n* = 6), AOM (10 mg/kg; *n* = 12), or AOM and waixA (*n* = 6) once a week for 3 weeks and sacrificed 2 weeks after the last injection. For waixA and its vehicle control, mice were given the i.p. injection 1 day before each AOM administration. Serum, urinary, fecal, and bone samples were collected for Mg^2+^ analysis (*p < 0.05 vs. vehicle). **e** Normalized average current development of human TRPM7 (hTRPM7) overexpressed in HEK293-TREx induced with tetracycline for 18–24 h [13] and assessed at +80 mV. Cells were either pre-incubated for 30 min. with 1 μM AOM and continued presence of AOM in the bath during the experiment (*n* = 6), or not (control; *n* = 4), or 1 μM AOM was acutely applied in standard external bath solution as indicated by the *black bar* (*n* = 3). **f** Average ACF-formation (*filled circles*) and colon length (*filled triangles*) in mice treated with vehicle, waixenicin A (8 mg/kg), or in combination with AOM (10 mg/kg; *n* = 4–6). Measurements are from mice in (**a**-**d**). *Error bars* indicate S.E.M. *Open circles* represent data points from individual animals. **g** Mg^2+^ concentrations in serum from control (*n* = 4) and AOM-treated (10 mg/kg; *n* = 10) mice fed with the control (0.05% Mg^2+^) or the Mg^2+^-deficient (0.003% Mg^2+^) diet sacrificed 2 weeks after the last of three AOM injections. Mice were kept on the control or Mg^2+^-deficient diet for 2 week before AOM injections. **h** Average ACF-formation (*filled circles*) and colon length (*filled triangles*) in mice from panel **f** (*p < 0.05 vs. AOM; *n* = 3–9). *Open circles* represent data points from individual animals

The results show that mice in the AOM group exhibited higher levels of Mg^2+^ concentration in feces (*p* < 0.05; Fig. 6b), indicating increased Mg^2+^ wasting in the colon through this agent. AOM treatment also led to higher serum Mg^2+^ levels (Fig. 6c) without affecting Mg^2+^ content of bone (Fig. 6d) or urine (Fig. 6a). The latter is independent of TRPM7, since neither 1 μM AOM pre-incubation between 30 min and 60 min nor acute application thereof inhibited overexpressed hTRPM7 currents as assessed by whole-cell patch clamp technique in tetracycline-inducible HEK293-TREx cells [13] (Fig. 6e). Finally, ACF formation was detected in the colons of all mice treated with AOM (Fig. 6f, left axis), but not in the colons of mice treated with vehicle control. AOM had no effect on colon length (Fig. 6f, right axis). We conclude that AOM treatment induces ACF formation and partially disrupts Mg^2+^ homeostasis independently of TRPM7, particularly affecting fecal excretion and serum levels.

### Waixenicin a decreases intestinal Mg^2+^ absorption and reduces Mg^2+^ concentration in serum

We next analyzed the end-point effect of waixA on systemic Mg^2+^ homeostasis in the AOM model (Fig. 6a-d). Injection of waixA once per week for three weeks, as outlined above, resulted in significantly increased fecal Mg^2+^ wasting accompanied by a strong reduction in serum levels (Fig. 6b, c), while neither influenced bone nor urine excretion. On the other hand, waixA had no further affect in AOM treated mice. Here, fecal Mg^2+^ levels reverted back to PBS control (Fig. 6b), while having no additional effect on serum Mg^2+^ levels compared to AOM (Fig. 6c). Thus, treating mice at lower levels with waixA once a week for three weeks induces hypomagnesemia through perturbed Mg^2+^ absorption, which is partially overwritten by concomitant AOM treatment. Finally, ACF formation and colon length was not affected by waixA treatment (Fig. 6f). We conclude that administration of waixA in vivo interferes with Mg^2+^ homeostasis, which is dependent the mode of delivery and point of analysis. We further conclude that using the current experimental design, waixA does not protect from the formation of chemically-induced pre-neoplastic lesions of adenocarcinoma.

### Formation of dysplastic aberrant crypt foci is independent of Mg^2+^ status in vivo

The role of Mg^2+^ in carcinogenesis remains a matter of debate [8], although solid tumor growth has been inversely linked to hypomagnesemia in mice [4]. Since short-term but repetitive treatment with waixA mimicked mild hypomagnesemia without preventing ACF formation (Fig. 6a-d and f), we wondered whether diet-induced severe hypomagnesemia would have an impact on intestinal neoplasia. We again employed AOM injections to induce ACF, this time in mice that were fed either the control diet or the Mg^2+^-deficient (0.003%) diet. After 1 week on their respective diet, mice were injected with either PBS alone or in conjunction with AOM (10 mg/kg) once a week for 3 weeks. The mice were sacrificed 2 weeks after the last AOM injection. To confirm hypomagnesemia, serum samples were analyzed for Mg^2+^ levels. As expected, all mice in the two Mg^2+^-deficient diet groups developed a significant decrease of Mg^2+^ concentrations in serum (*p* < 0.001; Fig. 6g). Diet-induced hypomagnesemia had no effect on body weight (Table 1), colon length, and on its own did not cause ACF formation (Fig. 6h) However, diet-induced hypomagnesemia with AOM caused a significantly shortened colon (*p* < 0.05) compared to control, and, although not statistically significant (*p* = 0.2), tended towards increased ACF formation (Fig. 6h). Thus, hypomagnesemia does not seem to be a confounding factor in early colon cancer development, as assessed by the AOM animal model.

## Discussion

The role of ion channels in carcinogenesis is not well understood. This is partially due to the paucity of available mechanistic models that allow correlation between ion channel function, cell proliferation, and carcinogenesis in vivo [32]. The study of Mg^2+^ in carcinogenesis is confounded by the absence of a clearly defined mechanism underlying Mg^2+^ regulation. Hence, few studies have addressed the effect of systemic Mg^2+^ availability on carcinogenesis or tumor progression. We identified TRPM7 as the molecular mechanism by which systemic Mg^2+^ homeostasis is regulated and developed a highly specific antagonist, waixenicin A, that suppresses growth of tumor cells in vitro [11, 21]. This enabled us to investigate the correlation between Mg^2+^ status and carcinogenesis in vivo based on a mechanistic model.

Little is known about the biophysical and pharmacological properties of TRPM7 and TRPM6 in cell lines of mammalian colon cancer or primary colon epithelial cells. We show that TRPM7 rather than TRPM6 plays a dominant role as a magnesium-transporting channel in HT-29 cells based on the following: [1]. By q-PCR, the mRNA expression of TRPM7 is much higher than TRPM6 (Fig. 1b); [2]. The IC_50_ for inhibition by intracellular Mg^2+^ is 600 μM for endogenous TRPM7-like currents in HT-29, and is more similar to hTRPM7 compared to hTRPM6 (Fig. 1d; [27, 29]); [3]. The endogenous TRPM7-like current is completely inhibited, rather than facilitated, by 2-APB (Fig. 1f; [26, 27]); [4]. 10 μM waixenicin A (waixA) significantly blocks the current in a Mg^2+^ dependent manner (Fig. 2; [21]); and [5]. TRPM6 siRNA had no effect on this current (Fig. 3b). Suppression of TRPM7 either by siRNA or inhibition by waixA inhibited cell proliferation in HT-29 cells. Unlike in chicken DT-40 B cells [13], high extracellular Mg^2+^ supplementation did not rescue cell proliferation in TRPM7-suppressed HT-29 cells despite comparable expression levels of alternate Mg^2+^ transporters (Fig. 3; [30]). Finally, we detected TRPM7-like currents in primary cultured mouse colon epithelial cells (MCECs), which were strongly inhibited by both 2-APB and by waixA in the presence of intracellular Mg^2+^ (Fig. 4). We cannot exclude that TRPM7 forms heteromeric ion channels with TRPM6 in HT-29 or MCECs, since heterologously overexpressed TRPM7/TRPM6 channels are have similar biophysical properties to TRPM7 [27].

Perturbed metal homeostasis, in particular excess of essential metals, has been associated with carcinogenesis and metastasis [33]. A meta-analysis revealed that Zinc (Zn^2+^) and copper (Cu^2+^) seem to play a particular role in head and neck cancer [34]. In HT-29 cells it is known that extracellular Zn^2+^ supports HT-29 cell growth at 10 μM and inhibits it at 100 μM through a Zn^2+^-sensing receptor that does not seem to alter intracellular Zn^2+^ concentrations and involves the ERK pathway [35]. Nickel (Ni^2+^) is typically associated with cell toxicity at various extracellular concentrations depending on cell type. For example, THP-1 cells, a monocyte-derived macrophage cell line, show strong cell growth suppression at 200 μM extracellular Ni^2+^ [36]. On the other hand, extracellular Ni^2+^ needs to reach 2 mM in order to perturb human osteosarcoma U2O2 or keratinocytes HaCat cell proliferation [37]. It will be interesting to decipher whether there is a connection between TRPM7, metal ions other than Ca^2+^ and Mg^2+^, and changes in cell proliferation.

Our data confirm that waixA inhibits native TRPM7 currents in both normal primary mouse colon epithelial cells (Fig. 4) and HT-29 colon adenocarcinoma cells (Fig. 2). Others showed that hypomagnesemia inhibits solid tumor growth and favors metastases in mice [4]. The influence of systemic Mg^2+^ deficiency on early stage carcinogenesis, however, remains controversial [8]. We here show that both, a single bolus injection and three weekly injections of waixA reduced Mg^2+^ levels in serum and altered Mg^2+^ absorption in colon (Figs. 5 & 6). Thus, waixA successfully induces hypomagnesemia, thereby implicating TRPM7 in the process. Azoxymethane (AOM) injections cause colonic neoplasia in rodents, mimicking the adenoma-carcinoma sequence seen in human patients [23]. Although mice fed with a diet deficient in Mg^2+^ developed hypomagnesemia, this neither exacerbated nor reduced ACF formation (Fig. 6). This was despite the drug’s effect to strongly reduce Mg^2+^ serum levels and decrease Mg^2+^ absorption in the colon, seemingly independent of TRPM7 (Fig. 6).

## Conclusion

We conclude that chemically-induced early stage colon cancer proceeds independent of systemic Mg^2+^ status and propose waixA as a pharmacological tool in the study of TRPM7 in vitro and in vivo. While ACF formation remains unaffected by waixA, its impact on actual tumor growth remains to be investigated.

## Additional file

Additional file 1: Cell Cycle Analysis. Description of data: Waixenicin A results in cell growth arrest in HT-29 cells as determined by cell cycle analysis using flow cytometry. (DOCX 613 kb)

## Abbreviations

2-APB: 2-Aminoethoxydiphenyl borate
ACF: Aberrant Crypt Foci
AOM: azoxymethane
BAPTA: 1,2-bis(*o*-aminophenoxy)ethane-*N*,*N*,*N′*,*N′*-tetraacetic acid
Ca^2+^: current-voltage (I/V); calcium
cDNA: complementary deoxyribonucleic acid
CNNM1: Cyclin and CBS domain divalent metal cation transport mediator 1
CNNM2: Cyclin and CBS domain divalent metal cation transport mediator 2
DMEM: Dulbecco’s Modfied of Eagle’s Medium
HEPES: *4-(2-hydroxyethyl)-1-piperazineethanesulfonic acid*
ICPOES: inductively coupled plasma optical emission spectrophotometer
MagT1: Magnesium Transporter 1
MCECs: Primary mouse colon epithelial cells
Mg^2+^: Magnesium
mRNA: messenger RNA
N33: Tumor suppressor candidate 3
NaOH: sodium hydroxide
NIPA1: non-imprinted in Prader-Willi/Angelman syndrome region protein 1
PBS: phosphate bufferd saline
PCR: Polymerase Chain Reaction
q-PCR: quantitative PCR
SEM: standard error of the mean
siRNA: small interfering ribonucleic acid
SLC41A1: Solute Carrier Family 41 Member 1
SLC41A2: Solute Carrier Family 41 Member 2
SLC41A3: Solute Carrier Family 41 Member 3
TRPM6: Transient Receptor Potential Member 6
TRPM7: Transient Receptor Potential Member 7
waixA: waixenicin A
