# Erratum to: Inhibition of TRPM7 suppresses cell proliferation of colon adenocarcinoma in vitro and induces hypomagnesemia in vivo without affecting azoxymethane-induced early colon cancer in mice

**DOI:** 10.1186/s12964-017-0188-8

**Published:** 2017-09-11

**Authors:** Junhao Huang, Hideki Furuya, Malika Faouzi, Zheng Zhang, Mahealani Monteilh-Zoller, Kelly Galbraith Kawabata, F. David Horgen, Toshihiko Kawamori, Reinhold Penner, Andrea Fleig

**Affiliations:** 1 Center for Biomedical Research, The Queen’s Medical Center, John A. Burns School of Medicine, University of Hawaii, 1301 Punchbowl St, Honolulu, HI 96813 USA; 2 Cancer Biology Program, University of Hawaii Cancer Center, 701 Ilalo St, Honolulu, HI -96813 USA; 3 Laboratory of Marine Biological Chemistry, Department of Natural Sciences, Hawaii Pacific University, Kaneohe, HI 96744 USA; 4 Present Address: Guangdong Provincial Key Laboratory of Sports and Health Promotion, Scientific Research Center, Guangzhou Sport University, Guangzhou, China; 5 Present Address: Department of Pharmacology, School of Pharmaceutical Sciences, Central South University, Changsha, Hunan China; 6 Present Address: Chikusa Central Clinic, Imaike, Chikusa-ku, Nagoya, Aichi Pref Japan

## Erratum

Unfortunately, after publication of this article [1], it was noticed that the names of Kelly Galbraith Kawabata and F. David Horgen were incorrectly presented as F. Kelly Galbraith Kawabata and David Horgen. The corrected author list can be seen above and the original article has been updated to reflect this change.
