# Supplementary material for: Inhibition of TRPM7 suppresses cell proliferation of colon adenocarcinoma in vitro and induces hypomagnesemia in vivo without affecting azoxymethane-induced early colon cancer in mice
Source: Cell Commun Signal. 2017 Aug 15;15:30. doi: 10.1186/s12964-017-0187-9 (PMC5558780; doi:10.1186/s12964-017-0187-9)
Supplement: Additional file 1: — Cell Cycle Analysis. Description of data: Waixenicin A results in cell growth arrest in HT-29 cells as determined by cell cycle analysis using flow cytometry. (DOCX 613 kb) [file 12964_2017_187_MOESM1_ESM.docx]

**Supplemental Information (SI)**

**Inhibition of TRPM7 suppresses cell proliferation of colon adenocarcinoma *in vitro* and induces hypomagnesemia *in vivo* without affecting azoxymethane-induced early colon cancer in mice**

Junhao Huang^1&*^, Hideki Furuya^2*^, Malika Faouzi^1^, Zheng Zhang^1,@^, Mahealani Monteilh-Zoller^1^, F. Kelly Galbraith Kawabata^3^, David Horgen^3^, Toshihiko Kawamori^2,#^, Reinhold Penner ^1,2^ and Andrea Fleig ^1,2^

^1^ Center for Biomedical Research, The Queen’s Medical Center, John A. Burns School of Medicine, University of Hawaii, Honolulu, HI-96813, U.S.A.

^2^Cancer Biology Program, University of Hawaii Cancer Center, 701 Ilalo St, Honolulu, HI-96813, U.S.A.

^3^Laboratory of Marine Biological Chemistry, Department of Natural Sciences, Hawaii Pacific University, Kaneohe, HI 96744, U.S.A

^&^Current Address: Guangdong Provincial Key Laboratory of Sports and Health Promotion, Scientific Research Center, Guangzhou Sport University, Guangzhou, China

^@^Current Address: Department of Pharmacology, School of Pharmaceutical Sciences, Central South University, Changsha, Hunan, China

^#^Current Address: Chikusa Central Clinic, Imaike, Chikusa-ku, Nagoya, Aichi Pref., Japan

*These authors contributed equally

**Cell cycle analysis**

Details of cell cycle analysis were previously described (20). Cell cycle analysis were determined by a FITC BrdU Flow Kit (BD Pharmingen) according to manufacturer’s instructions. Briefly, HT-29 cells were seeded at a density of 1 x 10^6^ cells/ml and allowed to equilibrate for 10 min before the addition of waixA. Cells were then incubated for another 20 min before BrdU was applied. After 6-hr incubation, the cells were fixed and permeabilized. DNase was added to expose the BrdU epitopes. Then the incorporated BrdU was stained with specific anti-BrdU fluorescent antibody. 7-AAD was used for staining of the DNA content. Cells were analyzed via flow cytometry (FACS, BD Biosciences) with excitation of 488 nm and FL-1 emission of 530 nm (bandpass filter) for FITC-conjugated anti-BrdU antibody detection and FL-3 emission of >670 nm (bandpass filter) for 7-AAD detection. The upper gate shows cells that have incorporated BrdU, including cells in the synthesis (S) and mitosis (M) phase of the cell cycle. The lower left gate shows dead cells and apoptotic cell debris (A). The middle lower gate represents cells in G1/G0-phase of the cell cycle (G1) where they have a normal DNA pool (2 n), whereas the right lower gate shows cells that have already doubled their DNA content (4 n) and are in G2-phase of the cell cycle (G2).

**Figure S1**


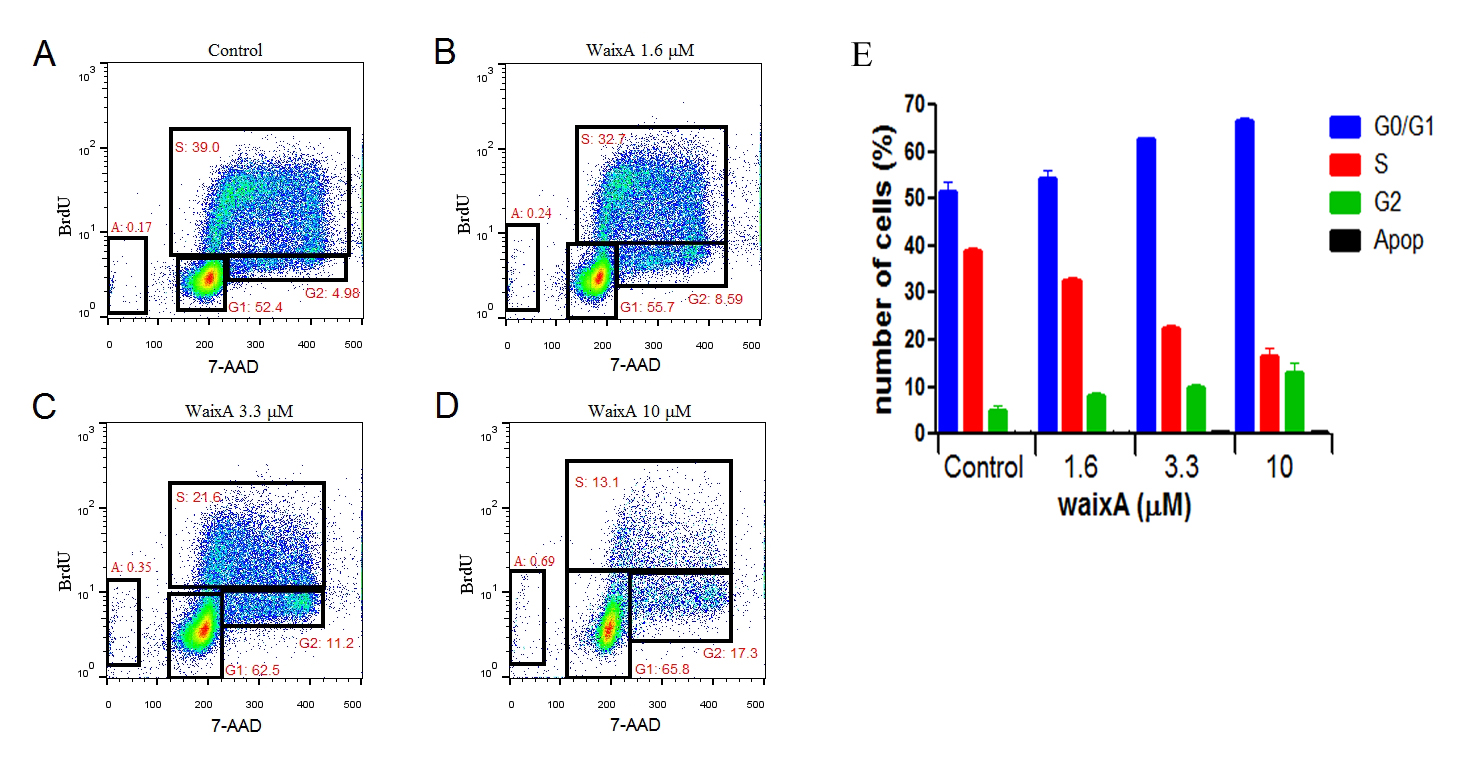


**Figure S1. WaixA results in cell growth arrest in HT-29 cells.** Cell cycle analysis of HT-29 cells incubated without (A) or with 1.6 μM (B), 3.3 μM (C) or 10 μM (D) of WaixA plus BrdU for 6 hr. Cells were stained with anti-BrdU antibody and 7-AAD and analyzed via flow cytometry. (E) Statistical analysis of the data set as in (A-D). Bars represent normalized cell numbers (n = 3).
